# α_1_-Microglobulin Protects Against Bleeding-Induced Oxidative Damage in Knee Arthropathies

**DOI:** 10.3389/fphys.2018.01596

**Published:** 2018-11-16

**Authors:** Staffan Larsson, Bo Åkerström, Magnus Gram, L. Stefan Lohmander, André Struglics

**Affiliations:** ^1^Department of Clinical Sciences Lund, Orthopaedics, Faculty of Medicine, Lund University, Lund, Sweden; ^2^Department of Clinical Sciences Lund, Infection Medicine, Faculty of Medicine, Lund University, Lund, Sweden

**Keywords:** α_1_-microglobulin, heme, hemoglobin, hemolysis, knee injury, knee osteoarthritis, oxidative stress, synovial fluid

## Abstract

Knee injury increases the risk of developing knee osteoarthritis (OA). Recent evidence suggests involvement of oxidative stress induced by inflammation and bleeding in the joint. This study investigates the role in this process of α_1_-microglobulin (A1M), a plasma and tissue antioxidant protein with reducing function, and heme- and radical-binding properties. We studied matched knee synovial fluid (sf) and serum (s) samples from 122 subjects (mean age 40 years, 31% females): 10 were knee healthy references, 13 had acute inflammatory arthritis (AIA), 79 knee injury 0–10 years prior to sampling, and 20 knee OA. Using immunoassays, we measured sf-A1M and s-A1M, sf-hemoglobin (sf-Hb), sf-total free heme (sf-Heme), and sf-carbonyl groups (sf-Carbonyl). We explored associations by partial correlation, or linear regression models with adjustments for age, sex and diagnosis, and evaluated diagnostic capacity by area under the receiver operator characteristics curve (AUC). The AIA group had 1.2- to 1.7-fold higher sf-A1M and s-A1M concentrations compared to the other diagnostic groups; other biomarkers showed no between-group differences. sf-A1M and s-A1M were with AUC of 0.76 and 0.78, respectively, diagnostic for AIA. In the injury group, the amount of bleeding in the joint was inversely correlated to time after injury when measured as sf-Heme (*r* = -0.41, *p* < 0.001), but not when measured as sf-Hb (*r* = -0.19, *p* = 0.098). A similar inverse association with time after injury was noted for sf-A1M (*r* = -0.30, *p* = 0.007), but not for s-A1M and sf-Carbonyl. Linear regression models showed that sf-Heme was more strongly associated with sf-A1M and sf-Carbonyl than sf-Hb. Independent of diagnosis, sf-Heme explained 5.7% of the variability in sf-A1M and 3.0% in the variability in sf-Carbonyl, but appeared unrelated to s-A1M. High sf-A1M and low sf-Heme or sf-Hb were independently associated with low sf-Carbonyl. In conclusion, our results demonstrate that independent of disease, Hb and heme within a knee joint correlates with an increased sf-A1M concentration that appears to be protective of oxidative damage, i.e., a reduction in carbonyl groups. High concentrations of A1M in synovial fluid and serum was further diagnostic for AIA.

## Introduction

Knee osteoarthritis (OA) is the most common joint disease and is a major cause of pain and disability in the older population worldwide (Liu et al., 2015). It is a complex and multifactorial disorder that represents a pathological imbalance of degenerative and regenerative processes involving the whole joint (Loeser et al., 2012). Obesity, genetic factors, and aging are some of the most prominent risk factors (Sharma et al., 2006), and in the younger population, previous knee injury is an important contributor to increased prevalence of knee OA – as much as half of those sustaining a knee injury in their 20s had developed post-traumatic knee OA in the following 12 to 14 years (Lohmander et al., 2004, 2007; von Porat et al., 2004).

Lately, much evidence has emerged to support a central role for participation of local inflammatory pathways in the disease processes (Scanzello, 2017), particularly so after knee injury (Goldring and Otero, 2011). As a part of the inflammatory response after injury, oxidative stress due to formation of reactive oxygen species (ROS) is involved both in direct damage of cartilage components and as integral factors in cell signaling leading to cartilage degradation (Henrotin et al., 2003). In addition to inflammation, cell-free hemoglobin (Hb) released via hemolysis is a potent inducer of oxidative stress. Spontaneous decomposition of Hb via auto-oxidation leads to the formation of ROS, free heme groups and free iron, which are highly reactive and have the ability to damage lipids, proteins, and DNA (Olsson et al., 2012).

Protection from Hb and heme toxicity exists on several levels, both by mechanisms removing cell-free Hb and heme, and by a complex network of antioxidant mechanisms that inhibit and eliminate the oxidative compounds and repair the oxidative damage caused. On the extracellular level, within the circulation, haptoglobin (Hp) and hemopexin (Hpx) are two of the most prominent scavenger proteins, with antioxidative properties through their capacity to remove cell-free Hb (by Hp) and heme (by Hpx). A major intracellular antioxidant is heme oxygenase-1 (HO-1) which, through its heme-degrading activity, plays a critical role in the protection of cells. However, during disease and damage these antioxidant defense systems are not sufficient to protect against harmful reactive compounds, damage to DNA, lipids and proteins arises. Oxidation of proteins result in stable carbonyl groups on amino acid side chains, and protein carbonyl content is one of the most widely used marker of protein oxidation and a general indicator of oxidative stress (Dalle-Donne et al., 2003).

Another protein that in the last decade has been shown to play a central role as a protective protein against oxidative stress is α_1_-microglobulin (A1M) (Åkerström and Gram, 2014). It is a 26 kDa plasma and tissue protein with the capacity to bind free radicals and heme groups, and the capacity to repair oxidative damage by chemical reduction. A1M is mainly produced in the liver, and a rapid flow of A1M through the blood and tissues binds harmful molecules and delivers them to the kidneys where they are degraded or excreted in the urine. In addition to the liver, smaller quantities are also expressed in most other cells in the body (Åkerström and Gram, 2014). Of particular interest for this investigation is that the expression of A1M has been shown to be up-regulated in several cells and tissues during oxidative stress conditions in response to elevated levels of ROS, Hb, and free heme (Olsson et al., 2007, 2011).

The main objective of this work was to study the role of A1M and bleeding-induced oxidative stress in the development of osteoarthritis after knee injury. Using a cross-sectional knee arthropathy cohort with matched serum and synovial fluid samples, we measured A1M and protein carbonyl groups as biomarkers of oxidative stress and Hb and free heme as biomarkers of bleeding, hypothesizing that increased A1M buffers against oxidative damage in synovial fluid.

## Materials and Methods

### Subjects and Samples

From a convenience cohort of previously studied individuals (Lohmander et al., 1989, 1999; Larsson et al., 2009; Kumahashi et al., 2015), we selected 122 subjects with matched knee synovial fluid and serum samples that were diagnosed as knee healthy references, having acute inflammatory arthritis (AIA) in the knee, knee injury (anterior cruciate ligament tear and/or meniscus injury), or knee osteoarthritis (Table 1). Diagnosis was made by arthroscopy, radiography, assessment of synovial fluid and clinical examination (Lohmander et al., 1989). Serum was prepared from whole blood that had been allowed to clot at room temperature (RT), with the clot removed by centrifuging at 1800 *g* for 10 min. Synovial fluid samples were aspirated without lavage, centrifuged at 3000 *g* for 10 min. Aliquots of serum and synovial fluid supernatants were stored at -80°C. All patient-related procedures were approved by the ethics review committee of the Medical Faculty, Lund University.

**Table 1:** Characteristics of the study subjects.

Study group	*n* (% women)	Age, mean years (*SD*, range)	Time after injury, range in weeks (years)
All subjects	122 (31)	40 (18, 14–86)	–
Reference	10 (10)	60 (11, 45–77)	–
Acute inflammatory arthritis^a^	13 (46)	63 (15, 29–81)	–
Injury^b^	79 (28)	31 (12, 14–69)	0–549 (0–10.5 years)
Osteoarthritis	20 (45)	51 (16, 24–86)	–

^a^*12 had pyrophosphate arthritis, and 1 Yersinia-triggered reactive arthritis.^*b*^12 had isolated anterior cruciate ligament (ACL) injury, 46 ACL injury with concomitant meniscus injury, and 21 had isolated meniscus injury*.

### α_1_-Microglobulin (A1M)-Concentrations

We used a radioimmunoassay (RIA) developed in-house for the detection of A1M in serum (s-A1M) and synovial fluid (sf-A1M) (Gram et al., 2015). Radiolabeling of A1M with ^125^I (Perkin Elmer Life Sciences) was done using the chloramine T method (Greenwood et al., 1963). Protein-bound iodine was separated from free iodide by gel-chromatography on a Sephadex G-25 column (PD10, GE Healthcare, Stockholm, Sweden). A specific activity of around 0.1–0.2 MBq/μg protein was obtained. The RIA was performed as described (Plesner et al., 1975). Briefly, goat antiserum against human A1M (diluted 1:6000) was mixed with ^125^I-labeled A1M (approximately 0.05 pg/ml) and unknown patient samples (serum, diluted 400x; synovial fluid, diluted 100x) or calibrator A1M-concentrations. After incubating overnight at RT, antibody-bound antigen was precipitated by adding bovine serum and 15% polyethylene glycol, centrifuged at 1600 *g* for 40 min, after which the ^125^I activity of the pellets was measured in a Wallac Wizard 1470 gamma counter (Perkin Elmer Life Sciences).

### Total Hemoglobin (Hb-Total) Concentrations

To determine the concentration of total Hb in synovial fluid (sf-Hb) we used a Human Hb ELISA Quantification Kit from Genway Biotech, Inc. (San Diego, CA, United States) according to the manufacturer’s instructions. Samples were analyzed diluted 1:2000, at which the lowest detection limit for samples was 12.5 μg/ml. Absorbance was read at 450 nm using a Wallac 1420 Multilabel Counter.

### Heme Concentrations

To determine the concentration of heme in synovial fluid (sf-Heme) we used QuantiChrom^TM^ Heme Assay kit (Gentaur BVBA, Brussels, Belgium; cat. no. DIHM-250) following instructions in the kit and diluting samples 10x with PBS.

### Protein Carbonyl Groups

Determination of the concentration of protein carbonyl groups in synovial fluid (sf-Carbonyl) was done by an ELISA, modified from the original description by Buss et al. (1997). Briefly, samples were diluted 4x in 6 M guanidine-HCl, 0.5 M K_3_PO_4_, pH 2.5, containing 10 mM DNP-hydrazine, incubated at RT for 45 min, mixed 1:200 with PBS and coated overnight in 96-well microtiter plates at 4°C. After washing and blocking for 1 h at RT with 0.1% reduced bovine serum albumin (BSA) in PBS, the plates were incubated with rabbit anti-DNP (Invitrogen, diluted 1000x) in PBS, 0.1% reduced BSA, 0.25% Tween-20 for 1 h at RT, washed and incubated 1 h at RT with horseradish peroxidase-conjugated porcine anti-rabbit IgG (Dako, diluted 2000x) in the same buffer. The wells were finally washed and developed by addition of a ready-to-use 3,3′,5,5′-tetramethylbenzidine substrate solution (TMB, Life Technologies, Stockholm, Sweden), stopping the reaction after 5 min by addition of 1 M HCl. The absorbance was read at 450 nm using a Wallac 1420 Multilabel Counter (Perkin Elmer Life Sciences, Waltham, MA, United States). Reduced BSA for blocking and incubation buffers, and oxidized BSA for standard carbonyl groups, were prepared as described (Buss et al., 1997). Measured concentrations are described as absorbance at 450 nm divided by total protein content of the sample (abs./tot. prot.). Total protein content in synovial fluid (diluted 10x) was determined with Pierce^TM^ BCA Protein Assay Kit (ThermoFisher Scientific, Inc.).

### Statistical Analysis

Synovial fluid samples with Hb concentrations below the lower limit of detection (12.5 μg/ml) were given the concentration 12.5 μg/ml. We created the ratio of A1M concentrations in synovial fluid to serum (A1M ratio sf/s) for evaluation of the relative influence of local versus systemic concentrations of A1M. Normal distribution was evaluated by visual inspection of histograms, normal q–q plots and box plots. Student’s *t*-test was used to compare A1M concentrations between sexes. For comparisons of biomarker concentrations between diagnostic groups we used univariate ANCOVA adjusted for age and sex with evaluation of residuals in histograms and normal p–p plots. Adjustments were included since groups were heterogeneous for these variables, and s-A1M was shown to increase with increasing age (Takagi et al., 1980), and to be higher in males compared to females (Takagi et al., 1980; Itoh and Kawai, 1990). For the same reason, we adjusted for age and sex in partial correlation analysis between biomarkers and time after injury (where longer time after injury was associated with higher age, *r* = 0.357, *p* = 0.001). Age, sex, and diagnosis was adjusted for in (a) linear regression models exploring joint bleeding (sf-Hb or sf-Heme) as explanatory factors for other biomarkers, and (b) linear regression models exploring the interaction of joint bleeding (sf-Hb or sf-Heme) and sf-A1M as explanatory factors for oxidative damage in the joint (sf-Carbonyl). In the linear regression models, sf-Heme, sf-Hb, sf-Carbonyl and the interaction variables ratio sf-A1M/sf-Heme and ratio sf-A1M/sf-Hb were log10 transformed to get linear relationships between variables, with evaluation of residuals in histograms and normal p–p plots.

In receiver operator characteristics (ROCs) analyses we evaluated A1M concentrations as a diagnostic tool for correctly diagnosing subjects with AIA. Evaluations were based on the area under the ROC curve (AUC) with cut-offs chosen to maximize the sum of the sensitivity and specificity.

We used SPSS version 24 for all statistical calculations, and made no adjustment for multiple comparisons due to the exploratory nature of the study.

## Results

### α_1_-Microglobulin by Diagnosis

**FIGURE 1:**
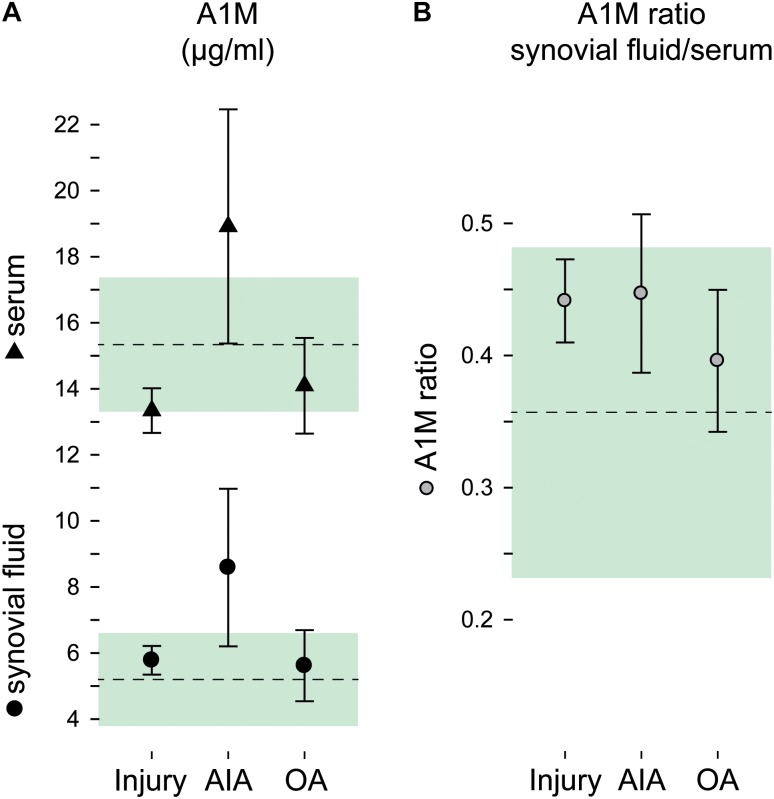
A1M by diagnostic groups. Concentrations of A1M in **(A)** synovial fluid and serum and **(B)** as a ratio in concentrations synovial fluid to serum. Group means and 95% confidence intervals are plotted as circles and triangles with error bars for the injury, acute inflammatory arthritis (AIA) and osteoarthritis (OA) groups, and as a dashed line and green area for the reference group. (Plotted data and statistics are reported in full in Supplementary Table S1).

α_1_-Microglobulin concentrations were normally distributed within the diagnostic groups, and were overall 2.6 times higher in serum compared to synovial fluid (Figure 1A), without difference between males and females (*p* = 0.80 and 0.62 for sf-A1M and s-A1M, respectively). The highest concentrations of A1M, both in synovial fluid and serum, were found in the AIA (Figure 1A). The between-group-differences were largest in synovial fluid, where the mean A1M concentration in the AIA group was 1.5-fold higher compared to the OA and knee injury groups (*p* = 0.005 and 0.045, respectively), while the 1.7-fold difference in means between AIA and the reference group failed to reach statistical significance (*p* = 0.068). In serum, the A1M concentration in the AIA group was between 1.3- and 1.4-fold higher compared to the injury and osteoarthritis groups (*p* = 0.035 and 0.030, respectively), whereas the 1.2-fold higher mean concentration compared to the reference group was not statistically significant (*p* = 0.15; Figure 1A).

The A1M ratio in synovial fluid to serum ranged between 0.36 and 0.45 in the four diagnostic groups, without statistically significant differences between groups (*p* = 0.36; Figure 1B).

Since the AIA group was standing out as the only group with increased A1M levels in synovial fluid as well as in serum, we performed a ROC curve analysis for having AIA using A1M in both fluids as the diagnostic tool. We found that with similar AUC close to 0.8, sf-A1M had higher specificity but lower sensitivity than s-A1M (Table 2).

**Table 2:** Receiver operator characteristic (ROC) curve analysis of sf-A1M and s-A1M as diagnostic for having acute inflammatory arthritis.

	sf-A1M	s-A1M
Cut off, μg/ml	7.625	15.4
AUC (95% CI)	0.763 (0.619, 0.908)	0.780 (0.648, 0.912)
sensitivity	0.615	0.692
specificity	0.844	0.734

*ROCs, receiver operator characteristics; AUC, area under the ROC curve*.

### Markers of Bleeding and Oxidative Damage

**FIGURE 2:**
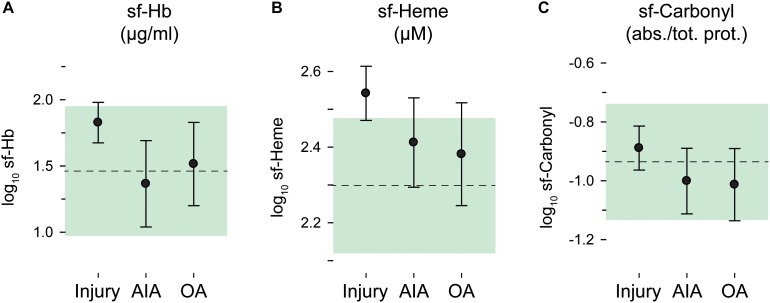
Oxidative stress in synovial fluid by diagnostic groups. Log_10_ transformed concentrations in synovial fluid (sf) of **(A)** hemoglobin (Hb), **(B)** total heme, and **(C)** carbonyl groups. Group means and 95% confidence intervals are plotted as circles with error bars for the injury, AIA and osteoarthritis (OA) groups, and as a dashed line and green area for the reference group. sf-Carbonyl values are given relative to total protein concentration. (Plotted data and statistics are reported in full in Supplementary Table S1).

Fifty-three out of 122 synovial fluid samples (43%) had sf-Hb concentrations below the lower limit of detection. Synovial fluid concentrations of Hb, total heme, and protein carbonyl groups were skewed, but were approximately normally distributed after log10 transformation. Intra-articular bleeding was more evident in the injury group, especially when measured as total heme (Figures 2A,B), but no statistically significant between-group differences were noted (*p* = 0.58 and 0.90 for sf-Heme and sf-Hb, respectively).

Oxidative damage in the joints, measured as protein carbonyl groups in synovial fluid, was highly similar and without statistically significant differences between diagnostic groups (*p* = 0.49) (Figure 2C).

### Temporal Variation in Biomarkers After Injury

**FIGURE 3:**
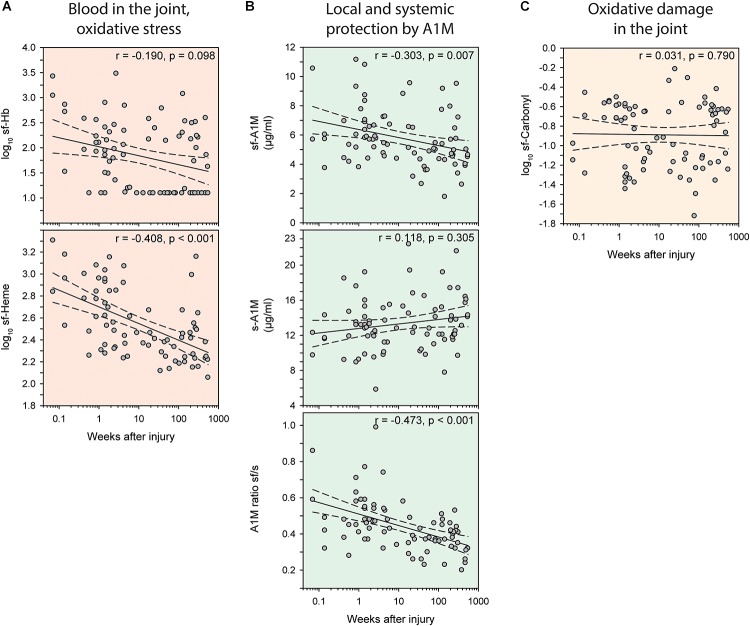
Temporal variations in biomarkers of oxidative stress after knee injury. Bivariate scatterplots of biomarker concentrations against weeks after injury with regression lines (solid) and 95% confidence intervals of the regression lines (dashed). (**A, red**) Blood in the joint measured as hemoglobin (Hb) or total heme in synovial fluid (sf). (**B, green**) Concentrations of A1M in synovial fluid and serum, and the ratio of A1M in synovial fluid to serum. (**C, yellow**) Oxidative damage measured as protein carbonyl groups in synovial fluid. Partial correlation coefficients (r) indicate correlations adjusted for age and sex.

In the injury group, the levels of the biomarkers of bleeding, Hb and heme, in the joint was highest early after injury, and longer times after injury were associated with less bleeding (Figure 3A). This inverse relationship was strongest when measured as sf-Heme, for which we (after adjusting for age and sex) observed a statistically significant inverse correlation with time after injury (*r* = -0.46), and less accentuated, and not statistically significant, when measured as sf-Hb (*r* = -0.19) (Figure 3A). A similar temporal pattern and inverse association with time after injury was noted for sf-A1M (*r* = -0.30) and for the ratio of sf-A1M/s-A1M (*r* = -0.47) but not for s-A1M and sf-Carbonyl (Figures 3B,C).

### Bleeding in the Joint as Explanatory Factor for Concentrations of A1M and Protein Carbonyl Groups Independent of Diagnosis

In hierarchical linear regression models including all 122 subjects, we examined how bleeding in the joint, independent of diagnosis, affected biomarker response after adjusting for age, sex and diagnostic group. This analysis showed that sf-Heme had a greater influence than sf-Hb on biomarkers of oxidative stress, since sf-Heme concentrations were associated with statistically significant effects on sf-A1M, the A1M ratio sf/s and sf-Carbonyl, whereas sf-Hb was only associated with an effect on the A1M ratio sf/s (Table 3). All associations had positive regression coefficients, i.e., higher concentrations of sf-Heme were associated with higher concentrations of sf-A1M, A1M ratios sf/s and sf-Carbonyl, and higher concentrations of sf-Hb were associated with higher A1M ratios sf/s (Table 3).

**Table 3:** Hierarchical linear regression models exploring joint bleeding as explanatory factors for other oxidative stress biomarkers in all 122 subjects with Heme or Hb entered last after age, sex, and diagnostic group.

.	log10 sf-Heme as explanatory						log10 sf-Hb as explanatory					
Dependent factors	^a^Effect, unstandardized (95%CI)	^b^Standardized effect	*p*-Value	Adjusted *R*^2^ for model	Adjusted *R*^2^ change when adding sf-Heme	Partial correlation	^a^Effect, unstandardized (95%CI)	^b^Standardized effect	*p*-Value	Adjusted *R*^2^ for model	Adjusted *R*^2^ change when adding sf-Hb	Partial correlation
sf-A1M	**2.169 (0.634, 3.705)**	**0.267**	**0.006**	**0.063**	**0.057**	**0.257**	0.420 (-0.250, 1.091)	0.118	0.217	0.009	0.005	0.114
Serum-A1M	1.934 (-0.272, 4.141)	0.150	0.085	0.227	0.013	0.163	0.263 (-0.674, 1.201)	0.047	0.579	0.206	-0.005	0.051
A1M ratio sf/serum	**0.121 (0.036, 0.207)**	**0.264**	**0.006**	**0.083**	**0.056**	**0.257**	**0.038 (0.001, 0.075)**	**0.190**	**0.045**	**0.046**	**0.025**	**0.185**
Log10 sf-Carbonyl	**0.214 (0.015, 0.414)**	**0.206**	**0.036**	**0.035**	**0.030**	**0.198**	0.082 (-0.002, 0.166)	0.183	0.056	0.026	0.023	0.176

^a^*Effect (regression coefficient): the estimate in average change in a dependent factor that corresponds to a 1-unit change in the explanatory factor log10-transformed sf-Heme or sf-Hb. ^*b*^*Standardized effect:* the estimate in average change in a dependent factor expressed in standard deviations that corresponds to a 1 standard deviation change in the explanatory factor log10-transformed sf-Heme or sf-Hb.*

*Numbers in bold face indicate statistically significance at the 0.05 level*.

The relative influence of sf-Heme and sf-Hb on sf-A1M, A1M ratio sf/s and sf-Carbonyl can be evaluated by comparing the standardized effect sizes, partial correlations and R^2^ changes in the models. These comparisons show that sf-Heme had the largest influence on sf-A1M and A1M ratio sf/s (with standardized effects and partial correlation coefficients of approximately 0.26), explaining 5.7 and 5.6% of the variability in sf-A1M and A1M ratio sf/s, respectively (*R*^2^ change = 0.057 and 0.056) (Table 3). The relative influence of sf-Heme on sf-Carbonyl was slightly lower (standardized effect size and partial correlation both close to 0.20), where the R^2^ change indicated that sf-Heme explained 3.5% of the variability of sf-Carbonyl in the model (Table 3). The lowest relative influence was noted for sf-Hb, which explained 2.5% of the variability of the A1M ratio sf/s (R^2^ change = 0.025, with standardized effect size and partial correlation below 0.2), without statistically significant influence on the individual concentrations of sf-A1M or s-A1M (Table 3).

### Interaction Between A1M and Hb or Heme as Explanatory Factors for Oxidative Damage Measured as Protein Carbonyl Groups Independent of Diagnosis

To investigate the combined effect of intra-articular bleeding and A1M on oxidative damage in the joint, we created two interaction variables, ratio sf-A1M/sf-Heme and ratio sf-A1M/sf-Hb. In two linear regression models, using heme as proxy for bleeding in model 1 and Hb in model 2, we then (in two steps, or blocks) explored the relationship between sf-Carbonyl and bleeding and sf-A1M alone (block 1) or in combination with the two interaction variables (block 2). Results in both models show that, when entered without an interaction variable, higher sf-Heme or sf-Hb were independently associated with higher oxidative damage measured as sf-Carbonyl (statistically significant positive effects), and higher sf-A1M was independently associated with lower sf-Carbonyl (statistically significant negative effects) (blocks 1, Table 4). Although different in direction, the relative influence of bleeding in the joint and sf-A1M on sf-Carbonyl was comparable in size (as judged by similarities in the magnitude of the standardized effects sizes and partial correlation coefficients in block 1 in both models) (Table 4). The model including sf-Heme and sf-A1M without an interaction variable explained 9.4% in the variability in sf-Carbonyl (adjusted *R*^2^ = 0.094) and the model including sf-Hb and sf-A1M explained 6.5% (Table 4).

**Table 4:** Linear regression models exploring the interaction of joint bleeding and A1M as explanatory factors for oxidative damage in the joint in all 122 subjects.

		Explanatory factors	Dependent factor: log10 sf-Carbonyl					
			^a^Effect, unstandardized (95%CI)	^b^Standardized effect	*p*-Value	Adjusted *R*^2^ for model	Partial correlation	Collinearity statistics, ^c^VIF
Model 1 Heme and A1M	Block 1	log10 sf-Heme	0.288 (0.088, 0.488)	0.277	0.005	0.094	0.263	1.2
		sf-A1M	-0.034 (-0.058, -0.010)	-0.267	0.005		-0.263	1.1
	Block 2	log10 ratio sf-A1M/sf-Heme	-1.549 (-0.392, -2.706)	-1.522	0.009	0.141	-0.246	44.1

Model 2 Hb and A1M	Block 1	log10 sf-Hb	0.094 (0.011, 0.178)	0.210	0.027	0.065	0.205	1.1
		sf-A1M	-0.028 (-0.050, -0.005)	-0.220	0.016		-0.222	1.0
	Block 2	log10 ratio sf-A1M/sf-Hb	-1.386 (-0.272, -2.500)	-3.106	0.015	0.104	-0.225	212.7

*Age, sex, and diagnosis were included as confounders in all models with Heme as proxy for bleeding in model 1 and Hb in model 2. In both models age, gender, diagnosis, sf-A1M and log10 sf-Heme or log10 sf-Hb was entered in the first block, with the interaction variable log10 ratio sf-A1M/sf-Heme or log10 ratio sf-A1M/sf-Hb entered together with the other variables in block 2.*

^a^*Effect (regression coefficient) the estimate in average change in the dependent factor log10 sf-Carbonyl that corresponds to a 1-unit change in the explanatory factors in the models.*

^b^*Standardized effect: the estimate in average change in the dependent factor log10 sf-Carbonyl expressed in standard deviations that corresponds to a 1 standard deviation change in the explanatory factors in the models.*

^c^*VIF (variance inflation factor): VIF higher than 10 indicates multicollinearity between variables*.

Adding the interaction variables (in block 2 of both models) increased the power of the models to explain variability in sf-Carbonyl from 9.4 to 14.1% when adding the ratio sf-A1M/sf-Heme, and from 6.5 to 10.4% when adding the ratio sf-A1M/s-Hb (Table 4). Based on the statically significant negative regression coefficients (unstandardized and standardized effects) (Table 4), higher ratios sf-A1M/sf-Heme or sf-A1M/sf-Hb were independently associated with lower concentrations of sf-Carbonyl.

When adding the interaction variables in block 2, multicollinearity between variables and their ratios was substantial in both models, as indicated by variance inflation factors (VIF) far beyond 10 (Table 4). This influenced the magnitudes of the effect sizes, making comparisons of effect sizes with and without the interaction variable present difficult. However, partial correlation coefficients for the interaction variables (ratios) and the individual concentrations were of the same magnitude (ranging between 0.20 and 0.26) (Table 4). This indicates that the relative influence on sf-Carbonyl of the ratio between sf-A1M and sf-Heme or sf-Hb is similar to that of the individual concentrations of sf-A1M, sf-Heme and sf-Hb.

## Discussion

This exploratory cohort study of oxidative stress in joint disease reveals a complex interplay between pro- and antioxidative molecular markers in knee injury, inflammation, or osteoarthritis. The study focuses on antioxidative reactions and protection from bleeding in the knee joint, and its main finding is that—without disease dependency—bleeding in the knee joint was associated with increased oxidative damage (in the form of higher concentrations of protein carbonyl groups in synovial fluid), as well as with higher concentrations of the antioxidative protein A1M. In addition, we saw that the net amount of oxidative damage was dependent not only on the absolute concentrations of Hb, heme or A1M, but that the ratios between pro- and antioxidative molecules were as important as their absolute concentrations. When adjusted for their respective absolute concentrations, the higher the proportion sf-A1M to sf-heme or sf-Hb, the less oxidative damage measured as protein carbonyl groups in synovial fluid. These novel data support a role for A1M in the protection against oxidative damage due to extracellular Hb and heme in the joint in various knee arthropathies.

The diagnostic group for which these findings may be of greatest value is the injury group, which had the highest synovial fluid concentrations of extracellular Hb and heme. For this group, we noted that the time-related pattern of cell-free Hb and heme in synovial fluid after injury was mimicked by a similar pattern of A1M in synovial fluid, whereas serum A1M and synovial fluid carbonyl groups appeared as unaffected by time after injury. Altogether, our best interpretation of these data is that increased levels of cell-free Hb and heme in synovial fluid early after injury triggered an increase in the synovial fluid A1M concentration that appeared to be protective of oxidative damage.

The ratio of A1M in synovial fluid to serum followed a similar temporal pattern after injury and was more strongly associated with time after injury than the synovial fluid concentration by itself. This provides support that the reaction to knee injury and/or cell-free Hb and heme is localized to the injured knee, rather than being a systemic response. Such a local response may be the result of an upregulated local production of A1M, similar to previous reports of an upregulation of the protein in liver, skin, placenta, retina, and blood cells upon oxidative stimulation (Olsson et al., 2007, 2011; Cederlund et al., 2013). However, it cannot be excluded that an increased influx of systemic A1M also contributes to the synovial accumulation of A1M, since (i) the overall concentration of A1M in serum is three times higher compared to synovial fluid and (ii) reports have shown that the permeability of the synovial membrane and its blood vessels of small molecules like A1M, is increased in the early phases after joint injury and by hemorrhage (Soren et al., 1978; Hettinga, 1979).

There are many examples of studies that have shown increased A1M in serum or urine to be a biomarker of diseases such as preeclampsia (Anderson et al., 2011, 2016; Gram et al., 2015), and kidney failure (Terzi et al., 2014; Kang et al., 2015). Our study extends those findings to include elevated A1M in both serum and synovial fluid as a biomarker of AIA, as suggested by the ROC-curves with AUCs close to 0.8. It has previously been speculated that the plasma concentration of A1M does not change during inflammation unless it is secondary to disorders affecting the liver and kidney functions in humans (Itoh and Kawai, 1990), or rats (Falkenberg et al., 1997). Although we have no record of illnesses secondary to the AIA that the subjects of our study were seeking health care for, and therefore cannot rule out involvement of kidney or liver disease in some of the subjects, we find it unlikely that illnesses secondary to the AIA would explain the elevations of A1M seen in these subjects at group level in both serum and synovial fluid.

Studies on A1M in synovial fluid are rare. The only two reports we find are proteomic studies of subjects with knee OA (Gobezie et al., 2007; Sohn et al., 2012). One of those studies reported presence of A1M without comparison to other diseases or references (Sohn et al., 2012), the other found A1M to be upregulated in synovial fluid in subjects with OA compared to normal synovial fluid (Gobezie et al., 2007). In similarity with both studies, we found detectable concentrations of A1M in synovial fluid in subjects with knee OA. However, we did not see different concentrations between healthy and osteoarthritic subjects as was reported by Gobezie et al. (2007), which may be due to differences in methodology (mass spectrometry versus RIA), in the selection of patients, or in the power between the studies.

Expression and synthesis of A1M has been shown to be upregulated in cells after exposure to heme and ROS (Olsson et al., 2007, 2011). This is consistent with our findings here that both heme and Hb were associated with increased levels of synovial fluid A1M. Thus, we may speculate that local production of A1M in the knee joint is induced by synovial fluid free Hb or heme. However, with A1M increased in both synovial fluid and serum in AIA, the present study further suggests that, in addition to heme and ROS, A1M may be regulated by inflammatory cytokines. In further corroboration for an inflammatory link, we note that the temporal pattern of synovial fluid A1M seen after injury in this cross-section study, is highly similar to findings in a longitudinal study of ACL injured subjects, in which synovial fluid levels of inflammatory cytokines were initially elevated with subsequent decreasing levels over 5 years (Struglics et al., 2015; Larsson et al., 2017). This type of regulation by cytokines has been proposed for another major scavenger of ROS, extracellular superoxide dismutase (EC-SOD or SOD3) (Strålin and Marklund, 1994, 2000).

α_1_-Microglobulin has been proposed as a therapeutic or diagnostic tool for several different conditions and diseases. The focus has so far mainly been on two areas of application: diagnosis and treatment of preeclampsia (Gunnarsson et al., 2017), and kidney protection in radiation therapy (Ahlstedt et al., 2015). The present study suggests that treatment with A1M following knee injury may prevent development of OA. In addition to the obvious protective benefit of exogenous A1M binding and neutralizing cell-free Hb and heme, it may also be beneficial as a repair mechanism in already damaged cartilage, since *in vitro* studies showed that collagen fibrils were restored to normal by addition of recombinant A1M after destruction already had begun (Olsson et al., 2011).

One of the novelties and strengths of this study—that we studied biomarkers of oxidative stress in matched samples of serum and synovial fluid—also proved to be a limitation, since it drastically reduced the number of available subjects to include from our convenience cohort of subjects with various knee-related illnesses. This may have increased the risk for spurious results, and reduced the likelihood of finding statistically significant differences between diagnostic groups. The study was further limited by its cross-sectional design, which reduced the possibility to draw firm conclusions on temporal change after injury since no repeated sampling was made within individual subjects. Lack of information on secondary or concomitant illnesses that may have influenced our results represents a further limitation. Finally, the presence of imputed values for sf-Hb may have influenced and/or concealed associations with time after injury, or with other biomarkers.

## Conclusion

**FIGURE 4:**
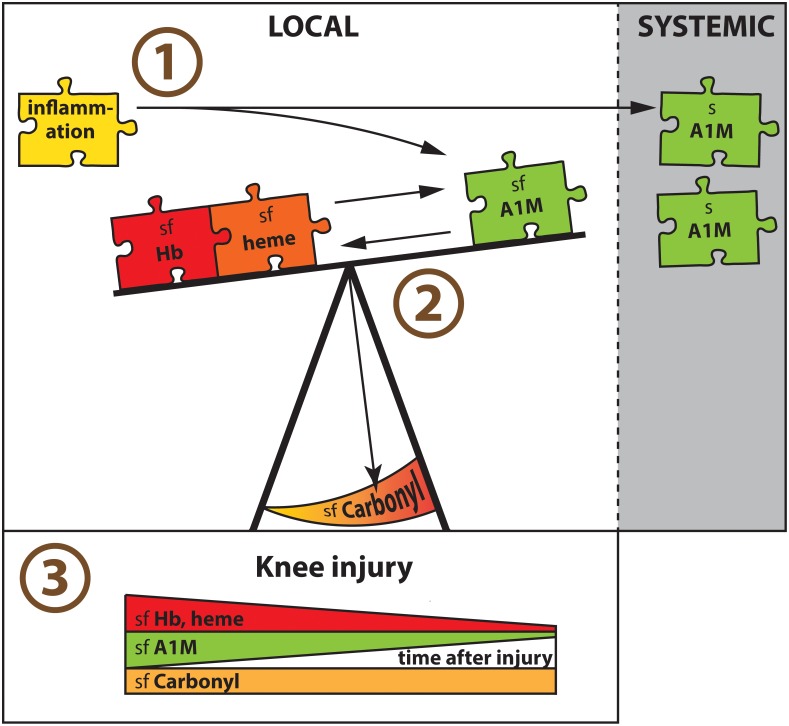
Major findings in summary. **(1)** Having AIA in a knee joint was associated with increased A1M concentrations locally in synovial fluid (sf) and systemically in serum (s). With area under the ROC curve of close to 0.8, A1M was a biomarker for AIA in both synovial fluid and serum. **(2)** Independent of disease, cell-free Hb and heme within a knee joint triggered an increased synovial concentration of A1M that appeared to be protective of oxidative damage measured as sf-Carbonyl. The net amount of local oxidative damage (sf-Carbonyl) was dependent not only on the absolute concentrations of oxidative Hb and heme, or antioxidative A1M, but the ratio between pro- and antioxidative molecules appeared as important. **(3)** In knee injured there was a similar temporal pattern in the synovial concentrations of both oxidants (Hb and heme) and antioxidants (A1M), which were highest early after injury, without a corresponding temporal change in oxidative damage (sf-Carbonyl).

Our results suggest (as summarized in Figure 4) that independent of disease, cell-free Hb and heme within a knee joint triggers an increased synovial concentration of A1M that appears to be protective of oxidative damage. We further conclude that the net amount of oxidative damage depends not only on the absolute concentrations of oxidative Hb and heme, or antioxidative A1M, but that the ratios between pro- and antioxidative molecules appears as important. Finally, we note that in AIA A1M is increased both locally in the joint fluid and systemically in serum, whereas in knee injury A1M is increased only in the joint fluid.

## Author Contributions

SL drafted the manuscript and full access to all of the data in the study and takes responsibility for the integrity of the data and the accuracy of the data analysis. SL, BÅ, LL, and AS were involved in study conception and design. BÅ and SL in the acquisition of data. All authors were involved in revising it critically for important intellectual content, approved the final version to be published, and the analysis and interpretation of data.

## Conflict of Interest Statement

BÅ and MG are co-founders and shareholders of A1M Pharma, Lund, Sweden. The remaining authors declare that the research was conducted in the absence of any commercial or financial relationships that could be construed as a potential conflict of interest.
