# Supplementary material for: α1-Microglobulin Protects Against Bleeding-Induced Oxidative Damage in Knee Arthropathies
Source: Front Physiol. 2018 Nov 16;9:1596. doi: 10.3389/fphys.2018.01596 (PMC6250851; doi:10.3389/fphys.2018.01596)
Supplement: Supplementary file 1 [file Data_Sheet_1.PDF]

α_1-microglobulin protects against bleeding-induced oxidative damage in knee arthropathies

Staffan Larsson, Bo Åkerström, Magnus Gram, L. Stefan Lohmander, André Struglics

Supplemental Table S1. Biomarker data by diagnostic group with statistics

s-A1M (µg/ml)	n	Mean	Median	SD	SE	95% CI of mean	Group comparisons by ANCOVA adjuted for age and sex, F (p-value)				
All subjects	122	14.22	13.35	3.79	0.34	13.54, 14.90	All 4 groups	vs Ref	vs AIA	vs Injury	vs OA
REF	10	15.34	15.25	2.84	0.90	13.31, 17.37	**3.91 (0.011)**	-			
AIA	13	18.92	17.40	5.87	1.63	15.37, 22.46		2.27 (0.15)	-		
Injury	79	13.34	12.90	3.02	0.34	12.66, 14.02		0.078 (0.78)	**4.60 (0.035)**	-	
OA	20	14.09	13.90	3.10	0.69	12.64, 15.54		0.065 (0.80)	**5.21 (0.030)**	0.43 (0.51)	-

sf-A1M (µg/ml)	n	Mean	Median	SD	SE	95% CI of mean	Group comparisons by ANCOVA adjuted for age and sex, F (p-value)				
All subjects	122	6.00	5.48	2.43	0.22	5.57, 6.44	All 4 groups	vs Ref	vs AIA	vs Injury	vs OA
REF	10	5.20	4.99	1.97	0.62	3.79, 6.61	**5.57 (0.001)**	-			
AIA	13	8.59	7.94	3.95	1.10	6.20, 10.97		3.74 (0.068)	-		
Injury	79	5.78	5.45	1.93	0.22	5.35, 6.21		0.021 (0.88)	**8.49 (0.005)**	-	
OA	20	5.62	4.68	2.30	0.51	4.54, 6.69		1.06 (0.31)	**4.41 (0.045)**	0.040 (0.84)	-

A1M Ratio sf/s	n	Mean	Median	SD	SE	95% CI of mean	Group comparisons by ANCOVA adjuted for age and sex, F (p-value)	
All subjects	122	0.43	0.41	0.14	0.012	0.40, 0.45	All 4 groups	*Post hoc analysis not applicable*
REF	10	0.36	0.28	0.18	0.056	0.23, 0.48	1.09 (0.36)	
AIA	13	0.45	0.43	0.099	0.027	0.39, 0.51		
Injury	79	0.44	0.42	0.14	0.016	0.41, 0.47		
OA	20	0.40	0.36	0.12	0.026	0.34, 0.45		

log10 sf-Hb (µg/ml)	n	Mean	Median	SD	SE	95% CI of mean	Group comparisons by ANCOVA adjuted for age and sex, F (p-value)	
All subjects	121	1.70	1.37	0.68	0.062	1.57, 1.82	All 4 groups	*Post hoc analysis not applicable*
REF	9	1.46	1.10	0.68	0.22	0.97, 1.95	0.19 (0.90)	
AIA	13	1.37	1.10	0.54	0.15	1.04, 1.69		
Injury	79	1.83	1.86	0.68	0.077	1.67, 1.98		
OA	20	1.51	1.10	0.67	0.15	1.20, 1.83		

log10 sf-Heme (µM)	n	Mean	Median	SD	SE	95% CI of mean	Group comparisons by ANCOVA adjuted for age and sex, F (p-value)	
All subjects	116	2.48	2.41	0.30	0.028	2.43, 2.54	All 4 groups	*Post hoc analysis not applicable*
REF	10	2.30	2.23	0.25	0.079	2.12, 2.48	0.66 (0.58)	
AIA	13	2.41	2.36	0.20	0.054	2.29, 2.53		
Injury	75	2.54	2.46	0.31	0.036	2.47, 2.61		
OA	18	2.38	2.30	0.27	0.064	2.25, 2.52		

log10 sf-Carbonyl (abs./tot.prot.)	n	Mean	Median	SD	SE	95% CI of mean	Group comparisons by ANCOVA adjuted for age and sex, F (p-value)	
All subjects	122	-0.93	-0.98	0.31	0.028	-0.98, -0.87	All 4 groups	*Post hoc analysis not applicable*
REF	10	-0.94	-1.04	0.28	0.087	-1.13, -0.74	0.81 (0.49)	
AIA	13	-1.00	-1.03	0.18	0.051	-1.11, -0.89		
Injury	79	-0.89	-0.80	0.33	0.038	-0.96, -0.81		
OA	20	-1.01	-1.03	0.26	0.059	-1.14, -0.89		

sf-Hb (µg/ml)	n	Mean	Median	SD	SE	95% CI of mean
All subjects	121	192.4	23.8	425.3	38.7	115.8, 269.0
REF	9	165.7	12.5	383.1	127.7	-128.7, 460.2
AIA	13	63.7	12.5	115.5	32.0	-6.1, 133.5
Injury	79	231.1	72.1	486.7	54.8	122.1, 340.1
OA	20	135.2	12.5	280.1	62.6	4.1, 266.2

sf-Heme (µM)	n	Mean	Median	SD	SE	95% CI of mean
All subjects	116	401.7	256.0	380.6	35.3	331.7, 471.7
REF	10	239.4	168.5	193.5	61.2	101.0, 377.8
AIA	13	283.8	227.0	131.7	36.5	204.8, 363.4
Injury	75	460.4	291.0	393.4	45.4	369.8, 550.9
OA	18	332.3	199.0	477.3	112.5	95.0, 569.7

sf-Carbonyl (abs./tot.prot.)	n	Mean	Median	SD	SE	95% CI of mean
All subjects	122	0.151	0.104	0.108	0.0098	0.132, 0.171
REF	10	0.140	0.092	0.089	0.028	0.075, 0.203
AIA	13	0.109	0.094	0.048	0.013	0.080, 0.138
Injury	79	0.168	0.160	0.117	0.013	0.142, 0.194
OA	20	0.118	0.094	0.095	0.021	0.074, 0.163
